# Living Arrangements, Electronic Communication Use, and Psychological Functioning

**DOI:** 10.1093/geroni/igaa057.331

**Published:** 2020-12-16

**Authors:** Hyunju Shim, Jennifer Ailshire, Eileen Crimmins

**Affiliations:** University of Southern California, Los Angeles, California, United States

## Abstract

Older people who live alone may benefit more from using electronic communication than those who live with others. Although living alone has been linked to a higher risk of depression and social isolation, few studies examined the effect of using electronic communication separately by living arrangements. The current study examines the effect of electronic communication use by living arrangements for people aged 65 and older. Using the 2011-2018 National Health and Aging Trends Study (NHATS), we examine how the frequency of emails/texts is associated with changes in psychological well-being and depressive symptoms accounting for sociodemographic, health, social network characteristics (N=6,897). Multilevel growth curve models showed that those living alone or with others were more likely to have fewer depressive symptoms at baseline if they used electronic communication, but the use did not affect their trajectory of depression. Those living alone or with others who used electronic communication did not have higher psychological well-being at baseline, nor did it affect their trajectory. The overall findings raise a question on the effectiveness of promoting electronic communication technology as a substitute for in person interaction for older adults living alone in the community.

